# Dental sectioning for intraoral equine cheek teeth extractions: 29 cases

**DOI:** 10.3389/fvets.2024.1367861

**Published:** 2024-02-15

**Authors:** Alexis Leps, Szabolcs Korsos, Michèle Clarysse, Lieven Vlaminck

**Affiliations:** Department of Large Animal Surgery, Anesthesiology and Orthopedics, Faculty of Veterinary Medicine, Ghent University, Merelbeke, Belgium

**Keywords:** equine, dentistry, teeth, extraction, sectioning

## Abstract

The objectives of this retrospective study were to describe cheek teeth extraction by the sectioning technique, the decision making to use this technique and its potentially associated complications. Sectioning for dental extraction purpose was used in 29/461 (6.3%) of cases. Oro-sinusal fistula was the main post-operative complication, with 4/29 (13.7%) cases developing a macroscopic communication between the alveolus of the tooth extracted and the adjacent sinus compartment. All teeth where sectioning was attempted were successfully extracted. Sectioning for dental extraction appears to be a safe technique that can be used instead of or in addition too other minimal invasive cheek teeth extraction techniques. Thorough preoperative planning including oroscopic examination and medical imaging modalities are required to help in decision making, as well as excellent sedation and analgesia and horse compliance.

## 1 Introduction

Dental extraction is commonly performed in equine patients to treat diseased teeth such as in the case of apical infections. Dental extraction techniques have evolved rapidly in the past decades, focusing on improved efficiency, and reduced collateral damage to surrounding tissues. The introduction of the dental oroscope as a perioperative tool (1), safer and easier standing neuroleptanalgesia, and new techniques such as minimal invasive transbuccal screw extraction (2) and partial coronectomy (3) have decreased the need for invasive dental extraction techniques and with it the risk of post-operative complications. Repulsion of diseased cheek teeth such as described by McIllwraight and Turner (4) and Coomer et al. (5) has been grossly abandoned due to its high rate of complications of up to 80% (6, 7). Dental sectioning is commonly used in small animal (8, 9) and human dental extraction (10) to facilitate removal of multirooted teeth. The technique has been utilized in equine patients to help in a variety of situations that do not allow easy extraction of cheek teeth (11, 12). The aims of this study were to describe the technique as it was used by the authors to facilitate extraction in complicated equine extraction cases, and to describe the associated complications that were encountered.

## 2 Materials and methods

### 2.1 Case records

The study design was a retrospective, descriptive, cohort case series. Clinical records of all equine cheek teeth dental extractions between October 2020 and July 2023 at the Department of Large Animal Surgery, Anesthesiology and Orthopedics of the Faculty of Veterinary Medicine, Ghent University were reviewed. Data obtained included anamnesis, patient sex, breed, age, clinical signs during examination, previous treatment attempts, and Triadan position of the extracted tooth. Long-term follow up was obtained by either routine reexamination at the clinic, or by a telephone questionnaire to the owner.

### 2.2 Clinical examination

Full clinical examination, including heart auscultation, temperature, and body condition score was performed on every patient at presentation, followed by systematic external palpation of the head and full oral examination using oral endoscopy (Karl Storz^®^ Dental Endoscope, Storz, Germany), (Medicam^®^ HD Camera, Medicam, Germany) under sedation with detomidine (Detogesic^®^, Zoetis, The Netherlands) at 0.015 mg/kg bwt and butorphanol (Torbugesic^®^, Zoetis, The Netherlands) at 0.15 mg/kg bwt.

### 2.3 Diagnostic imaging

Standard dental radiographic projections (left and right dorsal to ventral 30° oblique projections for maxillary cheek teeth; left and right ventral to dorsal 45° oblique projections for mandibular cheek teeth) were performed on every patient and assessed by an European College of Veterinary Medical Imaging diplomate. If radiographic images were not conclusive, a computed tomography (CTScan) examination of the horse's head was performed using a 320-slice scanner (Aquilion One^®^, Canon Medical Systems, Tokyo, Japan).

### 2.4 Anesthesia protocol for standing dental extraction

Dental extraction procedures were performed on standing sedated horses restrained in stocks. Horses were intravenously premedicated with acepromazine (Tranquinervin^®^, Dechra, UK) at 0.02 mg/kg bwt, followed by detomidine (Detogesic^®^, Zoetis, The Netherlands) at 0.015 mg/kg bwt and morphine (Sterop, Belgium) at 0.1 mg/kg bwt. Sedation was maintained with a constant rate infusion of detomidine at rates ranging from 0.01 to 0.02 mg/kg/h. Midazolam (Dormazolam, Dechra, UK) was used at doses of 0.01 mg/kg in bolus when masticatory movements hindered the feasibility of the procedure. Perioperative analgesia was provided with flunixine meglumine (Emdofluxine^®^, Emdoka, Belgium) at 1.1 mg/kg bwt. Perioperative antimicrobial coverage was provided with a single dose of Procaine Penicillin G (Penikel^®^, Kela, Belgium) at 22000 I.U/kg bwt until August 2022. No perioperative antimicrobial drugs were used after that date for dental extractions. Regional anesthesia was achieved by injecting 10 ml mepivacaine 2% (Mepidor^®^, Dechra, UK) around the maxillary (13) or infra-alveolar branch of the mandibular nerve (14) depending on the tooth to be extracted. In exceptional cases where the patient was not sufficiently cooperative to safely perform the extraction, the procedure was continued under general anesthesia. These patients were induced with Ketamine (Ketamidor^®^, Ecuphar, Belgium) at 2.2 mg/kg btw in combination with midazolam (Dormozolam^®^, Dechra, UK) at 0.6 mg/kg bwt. After intubation, anesthesia was maintained with a mixture of oxygen and isoflurane (Isoflutek^®^, Alvira, Spain).

### 2.5 Description of tooth sectioning technique

The horse's mouths were thoroughly rinsed with water until cleared of any food particles.

**Figure 1:**
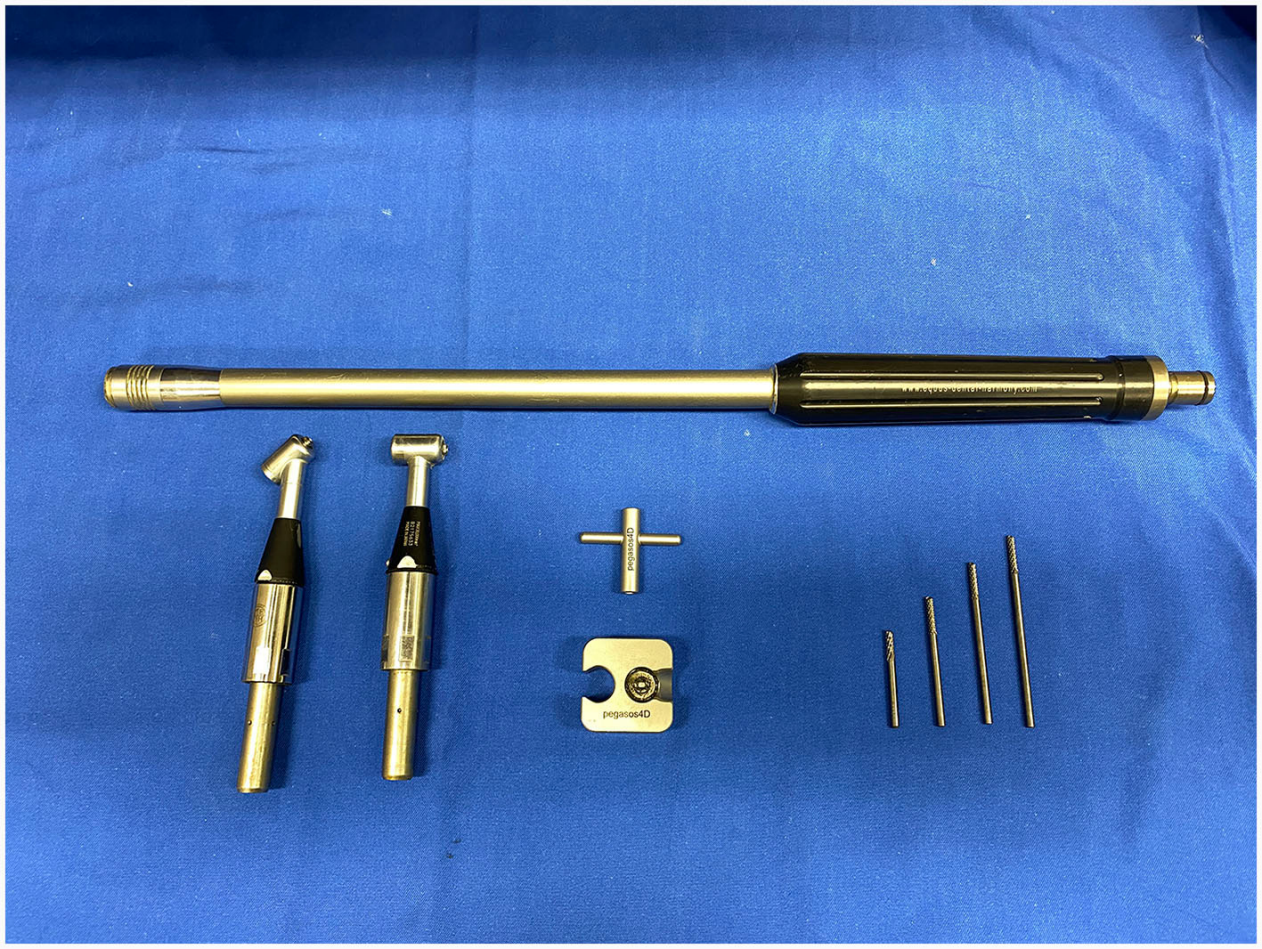
IC300 90° and KC300 50° heads mounted on the Equus Dental Harmony^®^ handle with 3.175 tungsten carbide sectioning burrs of 38-, 51-, 64-, and 77-mm lengths.

**Figure 2:**
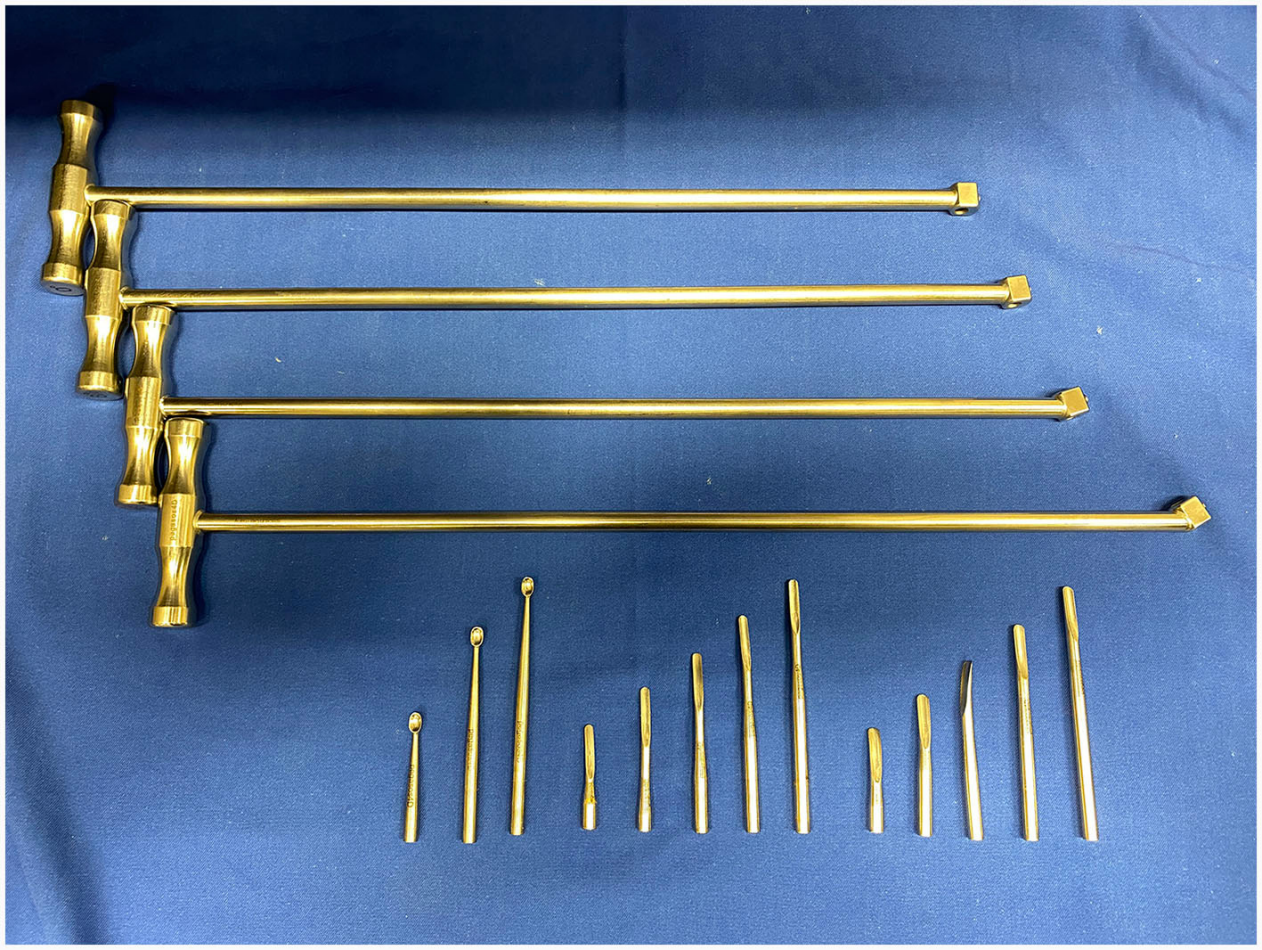
Elevator sets with 0°, 8°, 18°, and 30° T-handles with curettes, narrow (4 mm) and large (5 mm) elevators of variable lengths from Pegasos^®^.

Sectioning was performed with 3.175 mm diameter, double cut carbide burrs (Pegasos^®^, Germany) of different length (38, 51, 64, and 77 mm) mounted on an IC300 90° head (NSK, Japan) or a KC300 45° head mounted on a Equus Diastema 6000 extension (Equus Dental Harmony^®^, France) driven by an electrical motor (HDE, France) controlled by a pneumatic foot pedal switch. Oroscopic guidance and water-cooling were used throughout the procedure using an oroscopic irrigation sleeve (Storz^®^, Germany). This allowed to maintain a good visualization and to cool down the burrs and dental surfaces to avoid excessive heating of the tooth or surrounding tissues. Separated dental pieces were then elevated from the alveolus using a range of dental elevators (Pegasos^®^, Germany) (Figures 1, 2).

**Figure 3:**
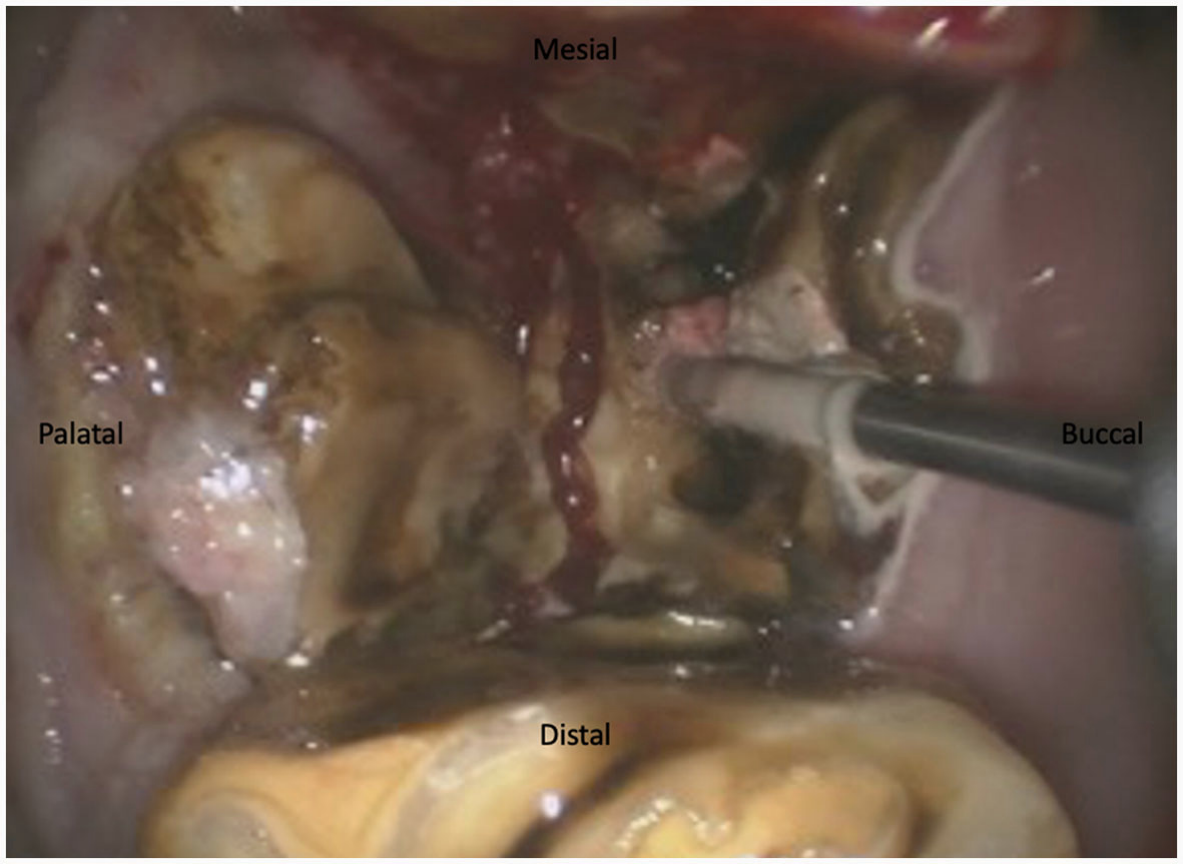
Illustration of a maxillary tooth 209 being sectioned in a 17-year-old Belgian Warmblood gelding. The first section was achieved in a mesiodistal direction. The burr is sectioning the buccal piece in two separate parts between the mesiobuccal and distobuccal root.

**Figure 4:**
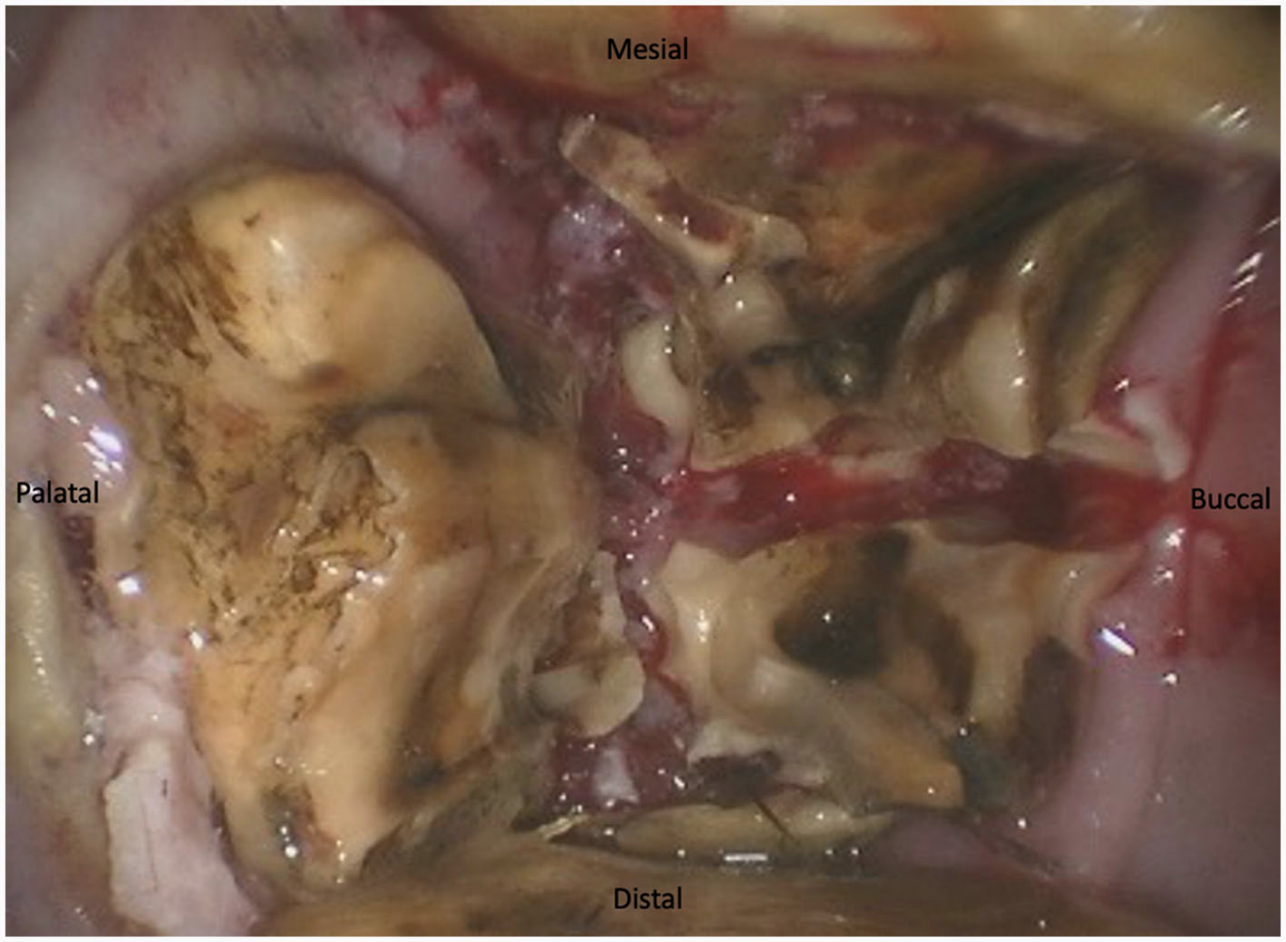
The tooth is now sectioned in 3 fragments that are ready to be elevated.

**Figure 5:**
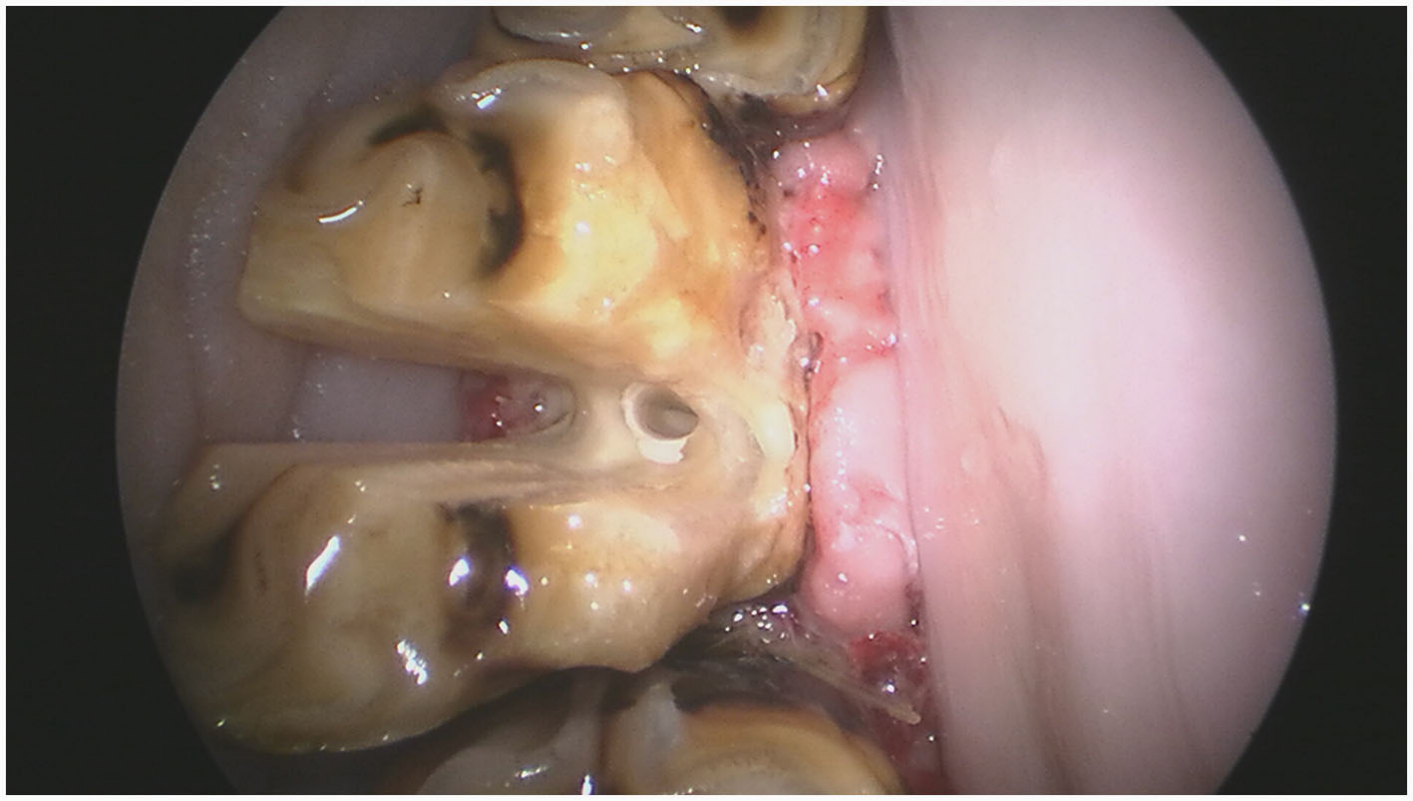
An intraoral endoscopic view of that same tooth illustrating the buccolingual cut halfway the clinical crown.

**Figure 6:**
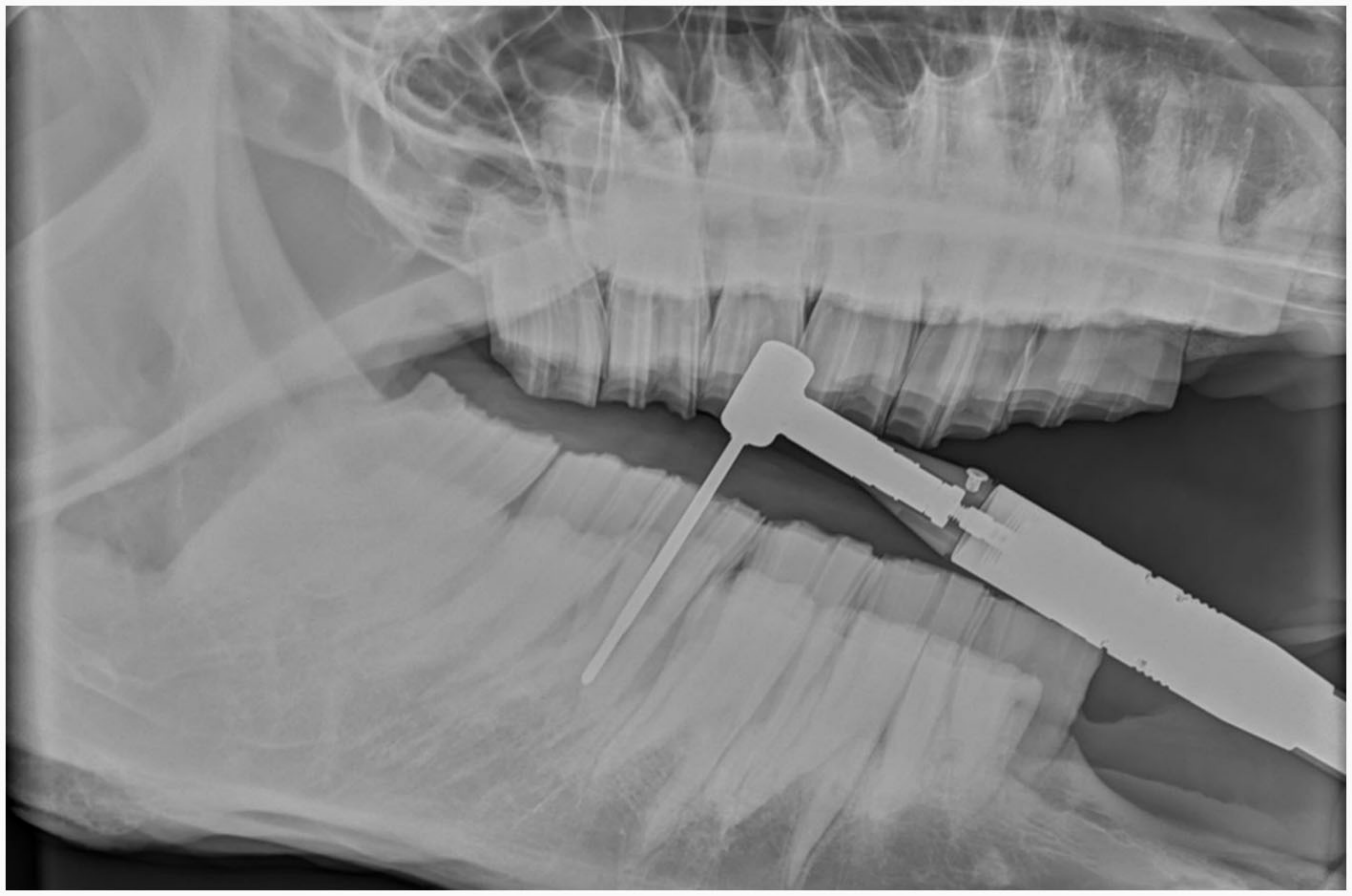
Intraoperative radiographic images help guide the trajectory of the burr while sectioning a mandibular tooth 309 in a 23-year-old warmblood mare.

For maxillary cheek teeth, the palatal root was separated from the buccal roots with a sagittal, mesio-distal cut in the crown using burrs of increasing length until bleeding periodontal ligament became visible in the cut through the entire mesio-distal width of the tooth (Figure 3). Once this cut was finished, luxation and removal of the dental fragments was attempted. If not successful, a second cut of the buccal piece was made in a transverse direction, aiming to separate the two buccal roots (Figure 4). Following removal of the different pieces, the alveolus was visually checked for any remaining fragments and thoroughly rinsed before applying polyvinyl siloxane (President Putty^®^, Coltene, Switzerland) to its oral aspect. Mandibular cheek teeth were sectioned halfway the mesio-distal length of the tooth in a linguo-buccal direction, aiming for the interradicular space. For these teeth radiographic guidance was used to ensure a proper trajectory of the cut as good visibility was harder to maintain because of fluids pooling in the alveolus due to gravity. When the cut was complete, further periodontal ligament luxation was performed and the two dental fragments were elevated one at a time (Figures 5, 6).

### 2.6 Decision making to section a tooth

**Figure 7:**
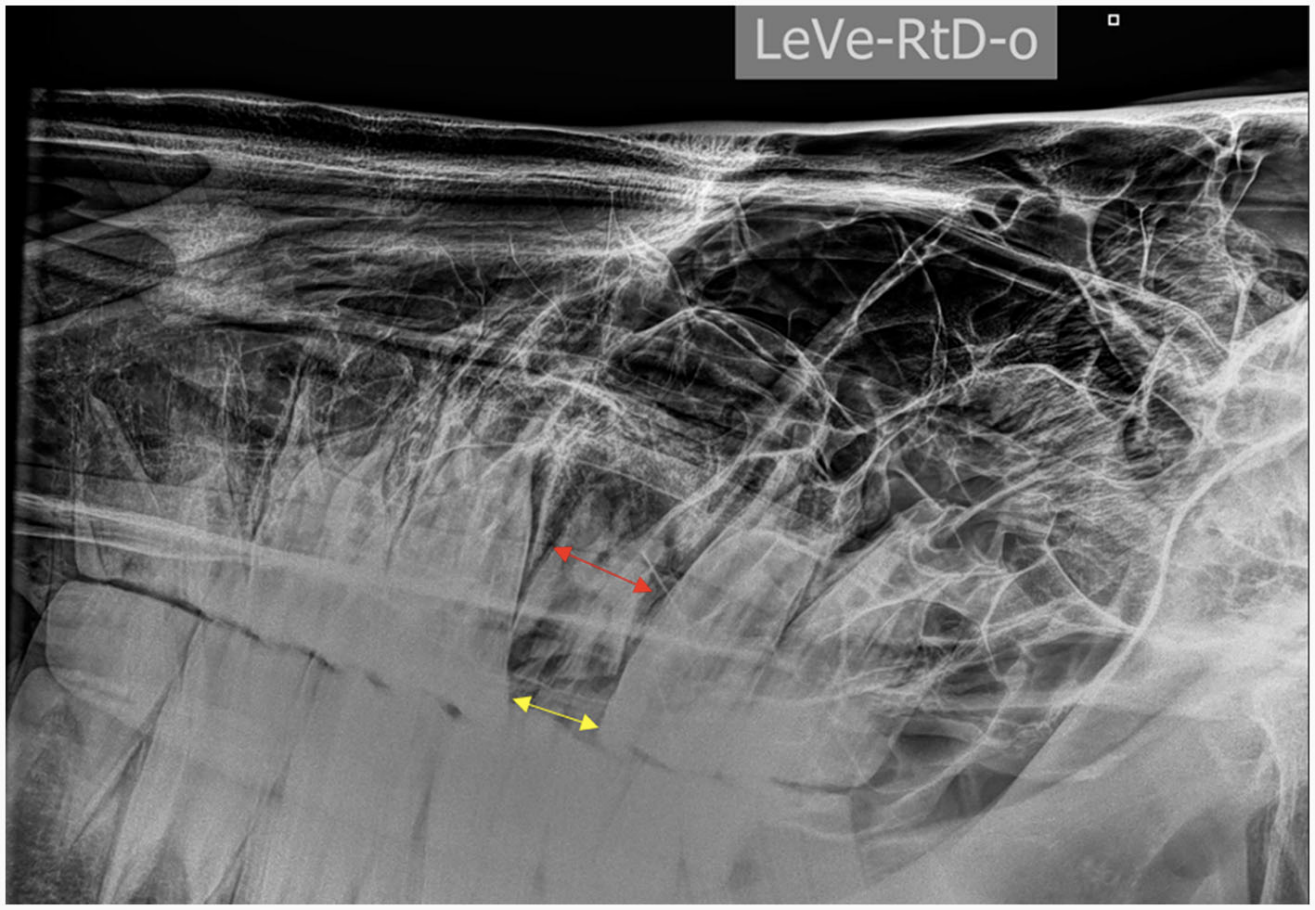
**Left** to **right** ventro-dorsal 30° projection of the head of a horse showing a chronically infected tooth 209 with limited space (yellow arrow) at the level of the occlusal surface compared to the apical mesiodistal width of the tooth (red arrow) due to dental drift following a complete fracture of the clinical crown.

- Scenario 1: If a tooth displayed very chronic disease, no clinical crown, and a drift of the adjacent teeth with contracture of the occlusal space, then the tooth was sectioned (Figure 7).
- Scenario 2: If a tooth was very decayed (multiple non-vital pulp exposures, advanced infundibular caries, and/or complicated clinical crown fracture) and during molar spreading or mobilization with molar forceps the clinical crown started to crumble and a partial coronectomy and/or minimally invasive transbuccal screw extraction (MITSE) technique could not be utilized, then the tooth was sectioned.
- Scenario 3: If a tooth presented with a normal clinical crown and good mobility after periodontal ligament manipulation with molar forceps but got stuck in the alveolus where avulsion forces were applied with a fulcrum, then MITSE technique was attempted. If MITSE technique was unsuccessful, then the tooth was sectioned.

## 3 Results

A total of 461 cheek teeth were extracted in 376 horses of which 292 (63.9%) and 169 (36.1%) were maxillary and mandibular cheek teeth, respectively. These horses included 49.1% mares, 50.3% geldings and 1.2% stallions with a mean age of 15.05 ± 6.02 years (range 2–30 years). If sufficient clinical crown was available, a standard extraction technique (15) was used to initiate the extraction process. In 356/461 (77.2%) cheek teeth this resulted in successful removal of the tooth. Depending on the individual situation and the surgeon's preference, the choice was made to redirect to more advanced extraction techniques in 105/461 (22.7%) teeth.

**Figure 8:**
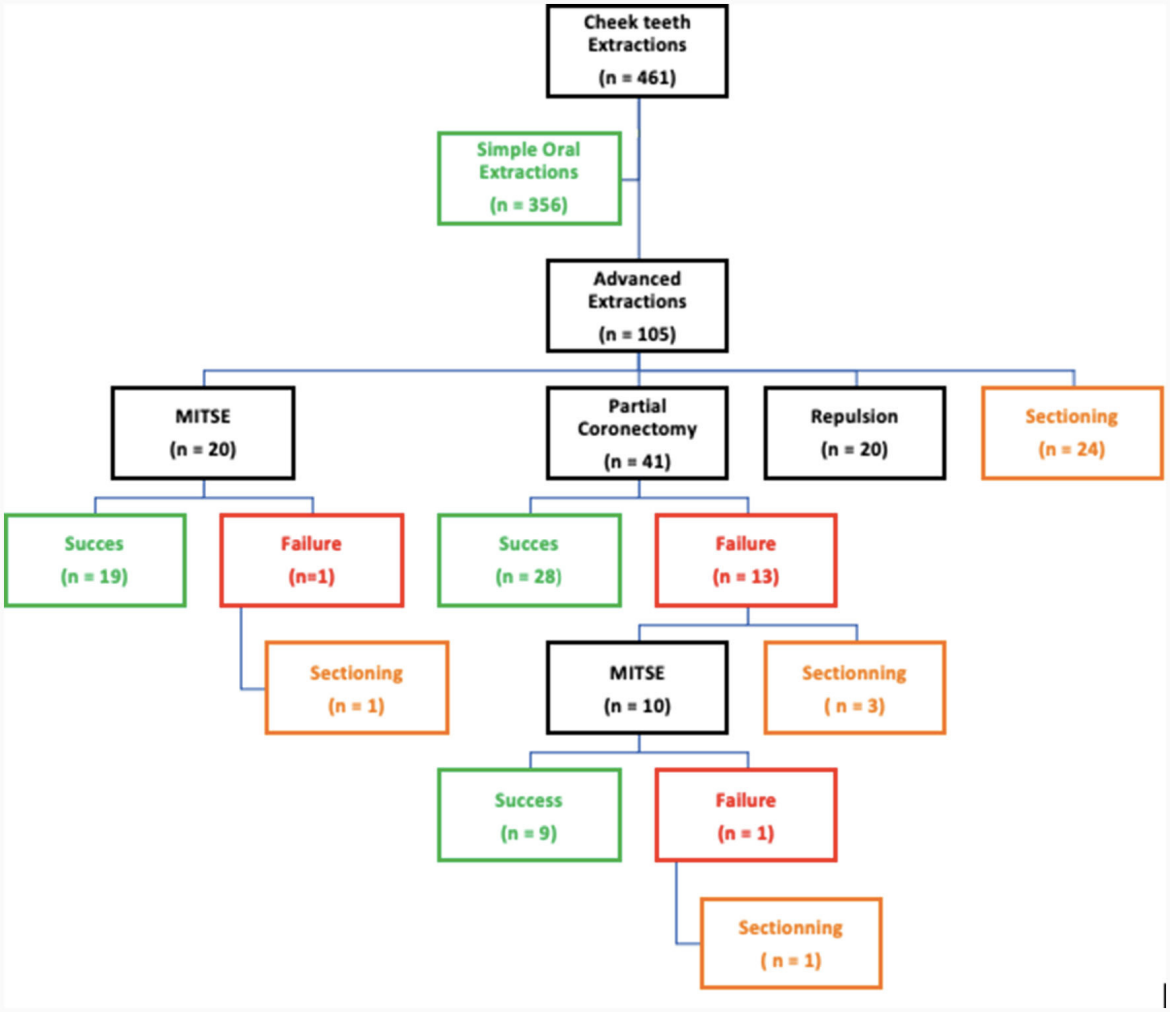
Changes of technique throughout the extraction procedure of 461 diseased equine cheek teeth.

Tooth sectioning was used to remove 29/461 (0.06%) teeth in 29/376 (0.08%) horses. Case details for these horses are summarized in Table 1. Most sectioned teeth were maxillary (27/29) with 14 of these sectioned into 2 pieces and 13 into 3 pieces to allow luxation. The main indication for these teeth to be extracted was chronic non-vital pulp exposure, with apical infection, with a loss of clinical crown integrity and interproximal drift of the adjacent teeth (Figure 8). The mean dental age of eruption of cheek teeth that were extracted by sectioning was 12.75 ± 4.2 (range 3–21).

**Table 1:** Case details for patients who underwent dental extraction by sectioning (NVPE, non-vital pulp exposure; CA/INF, infundibular caries; SFE, standard forceps extraction; OSF, Oro-sinusal fistula).

**No**	**Age (Years)**	**Triadan**	**Dental disease**	**First technique**	**Second technique**	**Third technique**	**N of pieces**	**Surgical time (min)**	**Complications**
1	7	109	Buccal slab fracture + NVPE 3, 4, 5	SFE (clinical crown crumbling at mobilization)	Sectioning	/	2	145	None
2	21	110	Palatal Slap Fracture + CA/INF/G4	SFE (Clinical crown crumbling at mobilization	Sectioning	/	2	30	None
3	20	110	Buccal Slap Fracture/NVPE 3, 4, 5	Partial Coronectomy mesial + SFE attempt (clinical crown crumbling at mobilization)	Sectioning	/	2	235	Peroperative perforation of sinus floor
4	13	209	Intradental fracture (Empty clinical crown/advanced decay)	Sectioning	/	/	3	45	None
5	19	309	Mobile tooth with dilacerated roots	SFE (no elevation possible despite good mobililty)	Sectioning	/	2	120	None
6	12	209	Complete clinical crown fracture + drift 208 – 210	MIB 3 times (dilacerated roots)	Sectioning	/	3	165	
7	10	110	Complete clinical crown fracture + drift 109 – 111	Sectioning	/	/	2	73	None
8	9	109	NVPE 1 to 5	SFE (clinical crown crumbling at spreading)	Sectioning	/	3	145	OSF
9	12	109	Buccal Slap Fracture + Attempted MIB	Sectioning (dilacerated palatal root)	/	/	3	90	None
10	10	209	Buccal Slap Fracture	Partial coronectomy distal + SFE	MIB	Sectioning	2	180	None
11	6	109	Sagittal fracture + enlarged reserve crown	Partial coronectomy distal + SFE attempt (clinical crown crumbling)	Sectioning + elevation fragments through buccotomy	/	2	90	None
12	17	209	Transverse fracture (mesial aspect absent)	Sectioning	/	/	3	175	None
13	14	109	Periapical abscess + enlarged roots	SFE, impossible to elevate	Sectioning	/	3	120	None
14	20	209	Complete fracture + drift 208 – 210	Sectioning	/	/	3	75	Perioperative Burr fracture
15	10	109	Microdontia + drift 108 – 110	Sectioning	/	/	2	75	Perforation Sinus Floor + OSF
16	5	109	Complete fracture after failed oral extraction, NVPE 1 to 5	Sectioning + elevetion fragments through buccotomy	/	/	3	80	Perforation Sinus Floor + OSF + sinusitis
17	12	109	NVPE 1 to 5	Partial Coronectomy mesial + SFE attempt (clinical crown crumbling	Sectioning	/	3	90	None
18	22	209	Complete fracture	Sectioning	/	/	2	35	None
19	19	207	Buccal Slap + Palatal Slap fracture	Sectionning	/	/	2	105	None
20	14	109	NVPE 1 to 5 + bad infundibula restaurations	SFE, crown crumbling,	Sectioning	/	3	185	None
21	12	210	CA/INF/G4 + NVPE 1 and 3	SFE, crown fracture at elevation despite satisfying mobility	Sectioning	/	2	90	None
22	17	111	NVPE 1 to 7 + advanced wear due to supraocclusion 411	Sectioning	/	/	3	120	None
23	15	209	Buccal slap + buccal infundibula fracture + NVPE 3, 4, 5 + Drift 208 – 209	Sectioning	/	/	2	40	None
24	4	209	Chronic NVT + empty crown + 7 cementomas	Sectioning	/	/	3	120	None
25	22	109	CA/INF/G3 mesial + NVPE 1 and 3	SFE, mesial aspect fracture at spreading	Sectioning	/	2	60	None
26	19	110	Complete fracture + 109 – 111 drift	Sectioning	/	/	2	150	None
27	21	110	Buccal slap + buccal infundibula fracture + NVPE 3, 4, 5 + Drift 109 – 111	Sectioning	/	/	2	120	None
28	13	109	Complete fracture + NVPE 1 to 5 + Drift 108 – 110	Sectioning	/	/	3	45	None
29	12	309	Complete fracture + Drift 308 – 310	Sectioning	/	/	2	150	Osteitis + Sequestra medial cortex mandibula

The mean surgical time to achieve total dental extraction for the 29 sectioning procedures was 106.6 ± 42 min (range 30–235). When sectioning was used as a 1st choice of advanced extraction technique, the mean surgical time of the procedure was 91.5 ± 33.2 min (range 35–175). In 3 horses the sectioning procedure had to be completed under general anesthesia due to either lack of cooperation of the patient despite deep sedation (2), or due to the impossibility to achieve sufficient analgesia (1).

Complications were recorded in a total of 7/29 (24.1%) cases. Intra-operative complications were encountered in 4/29 (13.8%) cases. In one (case 14) of these maxillary cases, the sectioning burr fractured but could be easily removed without further clinical consequences. In 3 maxillary cheek tooth cases, the floor of the overlying sinus compartment was perforated with the sectioning burr. Following standard alveolar sealing with polyvinyl siloxane (President Putty^®^, Coltene, Switzerland) for a duration of 2 weeks, one case (case 3) readily healed. In the two other cases (cases 15 and 16), at the first postoperative oral check-up at 2 weeks, the alveolus was seen to be partially granulated leaving a central oro-sinus fistula (OSF). Curettage of the fistulous tract and sealing with a polymethyl methylacrylate (PMMA) (Palapress^®^/Paladur^®^, Kulzer, Germany) plug at the oral aspect enabled uneventful healing in one of them (case 15) over a period of 4 weeks. The OSF persisted in the other horse (case 16) which subsequently developed chronic sinusitis of the rostral maxillary and ventral conchal sinus at 14 weeks post-surgery. This was treated by maxillary sinusotomy, and thorough curettage of the fistulous tract followed by sealing its oral aspect with PMMA. Complete healing was achieved 7 weeks after the revision of the OSF.

A post-operative curettage of the fistula 3 weeks after the revision was performed followed by sealing with a shortened PMMA plug. Postoperative complications were encountered in 3/29 cases. In two of these cases (cases 6 and 8) an OSF was identified at 2 weeks post-surgery while no communication between alveolus and sinus compartment was visible at oroscopic examination of the alveolus following complete removal of teeth at the time of the surgery. The OSF was not accompanied by any clinical sign suggestive of secondary infection of the overlying sinus compartments. In both cases, the fistulous tract was curetted, and the alveolus was routinely sealed with polyvinyl siloxane (President Putty^®^, Coltene, Switzerland) which resulted in spontaneous healing over 3 weeks. A third case (case 29) which involved sectioning of a mandibular 309 developed osteitis of the medial mandibular cortex. The mare developed a painful mandibular swelling 2 weeks post-operatively. Radiographic examination identified 3 bone sequestrums that required 2 curettages at an interval of 2 weeks each of the dental alveolus before it eventually healed after another 3 weeks.

## 4 Discussion

Dental sectioning has been reported in the literature (3, 12, 16) with little scientific data and description of the technique reported. This study reports the decision making to section a tooth, the surgical technique, and the peri and post-operative complications for 29 cases. In this study, most diseased equine cheek teeth (356/461, 77.2%) could be extracted using a standard oral extraction technique (15).

Standard oral dental extraction requires the presence of a healthy clinical crown that enables the use of molar spreaders and subsequent firm fixation of an extraction forceps. Dubois et al. (17) showed that cheek teeth with a clinical crown fractures were statistically less likely to be successfully extracted with simple oral extraction techniques and that the rest of individual CT configuration factors (dimension, type, triadan number, apical changes, infundibulum decay, root position, etc.) did not have an impact on the successful dental extraction rate. Other reasons that can impede straight-forward cheek tooth extraction, such as an increased diameter of the tooth's apical region or reserve crown due to dysplasia or chronic inflammation-induced peripheral cementum deposition, diverging roots (e.g., palatal root of a maxillary tooth), and impaction of a fractured tooth due to dental drift of adjacent teeth. However, correctly predicting if simple oral dental extraction will be successful remains difficult.

Preoperative planning based on a detailed oral examination supplemented by a radiographic and/or CT-scan evaluation of the diseased tooth is a prerequisite to allow proper decision making on which technique will be used. The aim of the surgeon should always be to work as minimally invasively as possible, preferably using an intra-oral approach that leads to fewer complications compared to more invasive dental extraction techniques such as transinusal repulsions (5, 7, 16, 18, 19). Based on the lack of progress during the procedure and any underlying complicating factors, one can change from one technique to another to successfully complete the extraction as was demonstrated in the present study (Figure 8).

Choosing between MITSE and segmentation should be a case-to-case decision, based on the configuration of the cheek tooth to be extracted, such as its length, the chronicity of the disease, the amount of dental decay, dental drift, enlarged reserve crown and/or roots. In exceptional cases, minimal invasive repulsion was used to remove a diseased cheek tooth in this case series. Transinusal maxillary cheek tooth repulsion was avoided at all costs. Some exceptional cases with severely dysplastic roots needed this approach to enable extraction.

Performing standing equine dental extraction requires very cooperative patients, even more during dental sectioning where the intra-oral use of high torque rotating carbide burrs can potentially cause iatrogenic collateral damage to soft and bony tissues if not completely controlled. The use of efficient neuroleptanalgesia techniques (20) and regional anesthesia of the maxillary or inferior alveolaris nerve techniques helped to make a tremendous progress in achieving successful dental extraction in the vast majority of patients in this study. However, general anesthesia could not be avoided in 3 cases where full cooperation of the horse was not achievable despite higher doses of sedation drugs and repeated regional anesthesia.

Water cooling is needed to keep the burrs clean of dental debris, so they are more efficient. Secondly it helps remove debris from the sectioning area, so it allows a better visibility and control on the depth of burring. Thirdly cooling also decreases friction which would cause heating of the tooth sectioned and surrounding tissues such as alveolar bone which could potentially result in bone sequestrums.

The main encountered complication in this case series was perforation of the sinus floor and/or development of OSF in 5/29 cases. Perforation of the apical alveolar bone easily results in communication with the adjacent sinus compartment. As mentioned above, water cooling helps visualizing the sectioning area and thus shows when the periodontal space has been reached as bleeding soft tissue will become exposed. Meticulous control of the sectioning instrument by the surgeon and optimal cooperation of the chemically restrained patient further contribute to avoiding unnecessary damage to the alveolar bone or perforating the sinus floor resulting in potential iatrogenic OSF. Perioperative radiographs for maxillary cheek tooth sectioning were not used in this case series. The authors emphasize radiographic guidance could help preventing iatrogenic sinus floor perforation. However, such guidance would not exclude the possibility of OSF development completely. In two other cases OSF developed postoperatively despite absence of intraoperative visible damage to the apical alveolar structures. Caramello et al. (7) reported that in 10% of extracted maxillary CT (Triadan 09 to 11) postoperative OSF developed regardless of the dental extraction technique used. Chronic periapical disease might cause disruption of the lamina dura weakening the floor of the adjacent sinus compartment thus rendering this area susceptible to collapse during dental extraction. Preoperative CT scan imaging might help to diagnose these preexisting lamina dura defects. However, the authors have experienced cases where absence of bone apically to the diseased tooth was diagnosed on CT scan images that did not develop OSF following tooth extraction. Further studies would be needed to be identify risk factors for development of postoperative OSF. Following tooth extraction, a minimum protective measure is to occlude the oral part of the alveolus with some kind of packing material to avoid food and increased bacterial contamination, and unnecessary pressure on the deeper alveolar tissues.

When perforation of the sinus floor (3 cases) occurred during the sectioning procedure, the post-operative management was not different from other cases. But owners were additionally asked to regularly observe their horse for development of nasal discharge in the ensuing weeks. Horses were seen at 2 weeks after the procedure to evaluate alveolar healing which was uneventful in two cases. In three cases that developed OSF, the problem was addressed by simple curettage to promote granulation and avoid epithelialization of the fistulous tract followed by routine sealing of the oral part. This resulted in rapid spontaneous healing in all but one case that required sinus surgery and repeated curettage before complete healing. In cases where OSF would become chronic, alternative techniques can be used such as summarized by Dixon (21).

Good visualization of sectioning depth in mandibular cheek teeth cases was not always achievable due to blood and fluids pooling in the sectioning area. This increases the likelihood of iatrogenic damage to surrounding structures. Using aspiration and radiographic guidance help directing the correct sectioning path between mesial and distal roots and to estimate sectioning depth. Regardless of the extraction technique used to extract a mandibular cheek tooth, more complications are to be expected compared to maxillary cheek teeth. In this case series, alveolar bone sequestration happened in 1/2 mandibular CT extracted by segmentation. Gergeleit and Bienert-Zeit (12) reported that mandibular alveolar bone sequestration was the most frequent complication following mandibular cheek tooth extraction representing 90% of the 6,6% of mandibular cheek teeth extraction complications.

The use of antibiotics in equine patients is subjected to constant debate whereas we, as practitioners, use too many antibiotics (22), possibly participating to the emerging multi resistant bacteria. It has been shown that transient bacteriemia occurs during dental extraction procedures as soon as gingival elevation was initiated in 90% of patients, with a total of 13 different bacteria genera identified (23), with most of these patients showing no symptoms of sepsis. Following Antimicrobial Stewardship in Equine Practice guidelines (22, 24), one should consider not using antibiotics in equine dental extractions as most cases do not develop systemic sepsis. Moreover, Christiansen et al. (25) has shown that the use of pre, peri, and post-operative antibiotics for cheek tooth extraction leads to the same rate of complications compared to not use antibiotics, regardless of the dental extraction technique used. The authors stopped using antibiotics after the results of this study were presented during the 31st ECVS annual scientific meeting in July 2022.

The present study showed that tooth sectioning is a good alternative technique to achieve dental extraction in cases that do not allow standard extraction techniques. The decision what alternative extraction technique to use is based on case specific details collected through clinical oral examination and medical imaging techniques. The complication rate was low in this case series but emphasized the need for meticulous control over the sectioning procedure and perfectly cooperative patients.

## Data availability statement

The original contributions presented in the study are included in the article/supplementary material, further inquiries can be directed to the corresponding author.

## Ethics statement

Ethical approval was not required for the studies involving animals in accordance with the local legislation and institutional requirements because surgery procedure had to be performed to alleviate pain. Written informed consent was not obtained from the owners for the participation of their animals in this study because it is a retrospective study, the technique does not need approval from the animal owners.

## Author contributions

AL: Conceptualization, Data curation, Investigation, Writing—original draft. SK: Methodology, Writing—review & editing. MC: Methodology, Writing—review & editing. LV: Supervision, Validation, Writing—review & editing.

## References

[1] Ramzan PHL Palmer L. Extraction of fractured teeth under oral endoscopic guidance in standing horses. Vet Surg. (2011) 40:586–9. 10.1111/j.1532-950X.2011.00804.x 21470251

[2] Langeneckert F Witte T Schellenberger F Czech C Aebischer D Vidondo B. Cheek tooth extraction via a minimal invasive transbuccal approach and intradental screw placement in 54 equids. Vet Surg. (2015) 44:1012–20. 10.1111/vsu.12409 26455833

[3] Rice MK Henry TJ. Standing intraoral extractions of cheek teeth aided by partial crown removal in 165 horses (2010–2016). Equine Vet J. (2018) 50:48–53. 10.1111/evj.12727 28744895

[4] McIllwraight C Turner A. Repulsion of cheek teeth. In:Hendrickson DA, editor. Techniques in Large Animal Surgery. 2 ed. Philadelphia, PA: Lea and Febiger (1989) p. 23–239.

[5] Coomer RPC Fowke GS McKane S. Repulsion of maxillary and mandibulary cheek teeth in standing horses. Vet Surg. (2011) 40:590–5. 10.1111/j.1532-950X.2011.00819.x 21466566

[6] Prichard MA Hackett RP Erb HN. Long-term outcome of tooth repulsion in horses. A retrospective study of 61 cases. Vet Surg. (1992) 21:145–9. 10.1111/j.1532-950X.1992.tb00033.x 1626385

[7] Caramello V Zarucco L Foster D Boston R Stefanovski D Orsini A. Equine cheek tooth extraction: comparison of outcomes for five extraction methods. Equine Vet J. (2020) 50:181–6. 10.1111/evj.13150 31260572

[8] Holmstrom SE Frost P Eisner ER. Exodontics. In: Veterinary Dental Techniques for the Small Animal Practitioner. 2 ed. Philadelphia, PA: Saunders (2004) p. 238–42.

[9] Reiter A. Closed and open tooth extraction. In: BSAVA Manual of Canine and Feline Dentistry and Oral Surgery. 4, ed. Gloucester: BSAVA (2018) p. 304–37. 10.22233/9781905319602.12

[10] Kaminishi RM Davis WH Nelson NE. Surgical removal of impacted mandibulary third molars. Dent Clin North Am. (1979) 23:413–25. 10.1016/S0011-8532(22)01208-3 288669

[11] Henry T Bishop I. Adjunct extraction techniques in equine dentistry. Vet Clin Equine. (2020) 36:565–74. 10.1016/j.cveq.2020.08.002 33067099

[12] Gergeleit H Bienert-Zeit A. Complications following mandibulary cheek tooth extraction in 20 horses. Front Vet Sci. (2020) 7:504. 10.3389/fvets.2020.00504 32923469 PMC7457057

[13] Staszyk C Biernet A Bäumer W Feige K Gasse H. Simulation of local anaesthetic nerve block of the infraorbital nerve within the pterygopalatine fossa: anatomical landmarks defined by computed tomography. Res Vet Sci. (2008) 85:399–406. 10.1016/j.rvsc.2008.02.008 18371997

[14] Tremaine W. Local analgesic techniques for the equine head. Equine Vet Educ. (2007) 19:495–3. 10.2746/095777307X207114

[15] Tremaine W. Oral extraction of equine cheek teeth. Equine Vet Educ. (2004) 19:495–503. 10.1111/j.2042-3292.2004.tb00287.x 15779621

[16] Kennedy R Reardon RJM James O Wilson C Dixon PM. A long-term study of equine cheek teeth post-extraction complications: 428 cheek teeth (2004 - 2018). Equine Vet J. (2020) 52:811–22. 10.1111/evj.13255 32144822

[17] Dubois B Dixon JJ Witte TH. Assessment of clinical and computed tomographic findings for association with the outcome of intraoral cheek tooth extraction in horses and ponies. J Am Vet Med Assoc. (2019) 255:1369–76. 10.2460/javma.255.12.1369 31793834

[18] Dixon PM Dacre I Dacre K Tremaine WH McCann J Barakzai S. Standing oral extraction of cheek teeth in 100 horses (1998 - 2003). Equine Vet J. (2005) 37:105–12. 10.2746/0425164054223822 15779621

[19] O'Neill HD Boussauw B Bladon BM Fraser BS. Extraction of cheek teeth using a lateral buccotomy approach in 114 horses (1999 - 2009). Equine Vet J. (2011) 43:348–53. 10.1111/j.2042-3306.2010.00169.x 21492213

[20] Schauveliege S Cuypers C Michielsen M Gasthuys F Gozalo-Marcilla M. How to score sedation and adjust the administration rate of sedatives in horses: a literature review and introduction of the Ghent Sedation Algorythm. Vet Anal Anesthes. (2019) 46:4–13. 10.1016/j.vaa.2018.08.005 30528671

[21] Dixon PM. Treatment of equine oro-nasal and oro-maxillary fistulae. Equine Vet Educ. (2020) 471–8. 10.1111/eve.13127

[22] Hardefeldt LY Bailey KE Slater J. Overview of the use of antimicrobial drugs for the treatment of bacterial infections in horses. Equine Vet Educ. (2021) 33:602–11. 10.1111/eve.13371

[23] Kern I Bartmann CP Verspohl J Rohde J. Biernet-Zeit A. Bacteraemia before, during and after tooth extraction in horses in the absence of antimicrobial administration. Equine Vet J. (2017) 49:178–82. 10.1111/evj.12581 27062656

[24] Raidal SL. Antimicrobial stewardship in equine practice. Austral Vet J. (2019) 97:238–42. 10.1111/avj.12833 31236925

[25] Christiansen MS Rosenmeier JG Jensen DB Lindegaard C. Standing equine cheek tooth extraction: a multivariate analysis of the effect of antibiotics on the risk of post-operative complications. Equine Vet J. (2023) 55:968–78. 10.1111/evj.13905 36516304

